# Attitudes Toward Alternative Tobacco and Nicotine Products and Their Association With Lifestyle Habits: Protocol for the MINERVA International Observational Cohort Study

**DOI:** 10.2196/67163

**Published:** 2025-10-14

**Authors:** Dario Gregori, Honoria Ocagli, Noor Muhammad Khan, Giulia Lorenzoni, Konstantina Thaleia Pilali, Ester Rosa, Francesca Angioletti, Aslıhan Şentürk Acar, Paola Berchialla, Danila Azzolina, Matteo Martinato

**Affiliations:** 1 Unit of Biostatistics, Epidemiology and Public Health Department of Cardiac Thoracic Vascular Sciences and Public Health University of Padova Padova Italy; 2 Department of Actuarial Sciences, Beytepe Hacettepe University Ankara Turkey; 3 Department of Clinical and Biological Sciences Torino University Torino Italy; 4 Department of Environmental Science and Prevention Ferrara University Ferrara Italy

**Keywords:** smoking, switch, survey, alternative tobacco and nicotine products, lifestyle

## Abstract

**Background:**

Traditional smoking is widely recognized as a public health issue, with established risks that include cardiovascular, respiratory, and cancer-related health impacts. Recently, alternative tobacco and nicotine products (ATNPs), such as electronic cigarettes, have gained popularity as potentially safer substitutes for traditional tobacco use. However, there remains uncertainty regarding the health effects of ATNPs, which require comprehensive research to evaluate their risks and potential benefits compared to conventional smoking.

**Objective:**

The aim of this multicenter observational study, MINERVA (My Changing Lifestyles Our Research and Everyone Health), is to examine the interplay between traditional smoking and the potential effect of switching to ATNPs in culturally diverse European countries.

**Methods:**

Data collection will be conducted through a structured web-based questionnaire and administered using a snowball sampling technique, which relies on the referrals and network connections of initial participants. A link will be provided for participants to access the questionnaire. Responders who express a willingness to participate in the study cohort will be recontacted at least twice a year to ensure continuity in their participation, and they will be asked to complete the same questionnaire during these follow-up assessments. This approach allows for the gathering of useful information over an extended period and the assessment of any changes or trends in the data that may emerge over time. Using social media for data collection, the research explores real-time attitudes and experiences related to smoking, with a particular focus on participants’ receptiveness to transitioning to nonconventional tobacco products. Statistical analysis will include descriptive statistics, and logistic regression models will examine associations. Nonlinear trends will be assessed using restricted cubic splines. Analyses will be conducted in R software with relevant statistical packages.

**Results:**

The MINERVA study began in June 2023, and data collection is still in progress. As of January 2025, 7535 participants had been enrolled in Italy, of whom 4862 have consented to a follow-up. Preliminary analyses are currently being conducted.

**Conclusions:**

The study underscores the importance of ongoing research to inform evidence-based policies and interventions, emphasizing the role of social media surveys in capturing diverse perspectives and facilitating longitudinal studies for a deeper understanding of the health implications of transitioning from traditional smoking to ATNPs.

**International Registered Report Identifier (IRRID):**

DERR1-10.2196/67163

## Introduction

Tobacco smoking is a well-known major global public health concern, with well-documented adverse effects on human health [1-6]. Traditional smoking involves inhalation of combusted tobacco, which releases several harmful chemicals, including nicotine, tar, carbon monoxide, and various carcinogens.

Exposure to traditional tobacco products is widely known to be associated with adverse health outcomes, including, but not limited to, cardiovascular diseases, respiratory diseases, cancer, and other health effects (eg, reduced fertility, low birth weight in infants of smoking mothers, increased risk of infections, and impaired wound healing).

In recent years, electronic cigarettes (e-cigarettes or vapes) introduced a new form of nicotine consumption known as e-smoking or vaping. E-cigarettes work by vaporizing a liquid (e-liquid) that typically contains nicotine, flavorings, and other chemicals. E-smoking emerged as an alternative to traditional smoking, often marketed as a less harmful alternative [7]. While e-cigarettes do not involve the combustion of tobacco, they are not without health risks. Studies have shown that e-cigarettes expose users to a different set of chemicals, which can lead to nicotine addiction, respiratory and cardiovascular health problems, and unknown long-term effects [8-10].

However, it is noteworthy that literature has suggested that there could be several advantages of alternative tobacco and nicotine products (ATNPs) over traditional ones. One of the primary advantages of transitioning to ATNPs is the potential for better nicotine dependency management [8]. E-cigarettes allow users to regulate nicotine intake through the choice of e-liquid nicotine concentration and the frequency of use. This can facilitate a gradual reduction in nicotine consumption, aiding smokers in the process of quitting altogether. Compared to traditional smoking, ATNPs significantly reduce the exposure to many harmful chemicals found in tobacco smoke [9]. E-cigarettes do not involve combustion, which eliminates the formation of tar, carbon monoxide, and several carcinogenic compounds. While e-liquids may contain some chemicals, their overall composition is less toxic than that of tobacco smoke. Studies often suggest that transitioning from traditional smoking to ATNPs may lead to improvements in respiratory health [10]. ATNPs also offer potential benefits for nonsmokers and bystanders. Unlike traditional smoking, which emits harmful secondhand smoke, e-cigarettes primarily release water vapor [11]. This reduces the risk of passive exposure to harmful chemicals, thus protecting the health of those around the e-smoker. Furthermore, transitioning to ATNPs may lead to immediate improvements in quality of life [12]. E-smokers often report enhanced taste and smell sensations, as well as reduced coughing and phlegm production. These subjective improvements contribute to an overall sense of well-being [13].

MINERVA (My Changing Lifestyles Our Research and Everyone Health) is an observational international study that encompasses four Mediterranean countries—Italy, Greece, and Turkey—each contributing a distinctive cultural, regulatory, and epidemiological backdrop for evaluating the interplay between traditional smoking and the potential advantages of transitioning to ATNPs.

The objective of the project is to provide a comprehensive profiling of individuals based on their sociodemographic characteristics, lifestyle behaviors, and dietary habits, investigating their association with ATNP use and willingness to switch to alternative nicotine products.

## Methods

### Study Design and Participants

An observational international cohort study will be conducted. A questionnaire will be administered using a snowball sampling technique, which relies on the referrals and network connections of the initial participants. Snowball sampling was selected due to its ability to efficiently recruit individuals engaged in smoking behaviors and those transitioning to alternative tobacco products, where social networks influence participation. This method also allows for streamlined recruitment without relying on preexisting registries [14].

To be eligible for inclusion, individuals must be aged between 18 and 99 years. This criterion ensures that the study population exclusively consists of adults, encompassing a broad spectrum of age groups, from early adulthood to the later stages of life. This diverse demographic range aims to capture a comprehensive representation of adult participants, allowing for a thorough examination of the intervention’s effects across various life stages. The study will be initially conducted in Italy and later in Turkey and Greece.

Participants who consent to be recontacted for inclusion in the cohort will undergo biannual assessments. To ensure continuity in the biannual assessments, participants who consent to follow-up will receive scheduled reminders via email, social media, or phone (if contact information is available). Additionally, engagement strategies such as periodic study updates will be implemented to encourage ongoing participation.

Individuals who face challenges in accessing and completing the questionnaire and those who experience difficulties reading or responding to the questionnaire will be excluded from participation. Additionally, individuals who are unable to accept the privacy and participation policy will not be eligible for inclusion in this study.

Participants who express their willingness to be recontacted will be part of the cohort for this study. The cohort group will be asked to complete a short version of the questionnaire during these follow-up assessments. This approach permits the gathering of valuable information over an extended period and assessing any changes or trends in the data that may emerge over time.

### Ethical Considerations

The study was carried out according to the Declaration of Helsinki guidelines and was approved by the Bioethics Committee of the University of Turin (protocol 0075071) on February 2, 2024.

All participants will provide informed consent before participation, with the option to withdraw at any time. Consent to participate and to collect and process personal data is given electronically (ticking the corresponding box) on the project website, after reading detailed information on data collection and processing, and before accessing the questionnaire, in accordance with the European Commission General Data Protection Regulation (679/2016).

Both the informed consent form for study participation and the data processing consent form have been approved by the institutional ethics committee.

Data will be fully anonymized to ensure privacy and confidentiality. No compensation will be provided for participation. No identifiable personal data will be included in the manuscript or the supplementary materials.

### Sample Size

A feasibility assessment was performed using a fixed model multiple regression and correlation analysis approach, parameterized according to a noncentral *F* distribution, which can consider the effect size of the population means. This accounts for differences in expected sample size (ie, a larger effect size requires a smaller sample size to achieve the same level of power). The noncentrality parameter (λ) has been established using the following equation:

λ = *f*^2^(u + v + 1)

Where u indicates the *df* for the numerator (ie, the number of independent variables) and v is the *df* for the denominator (ie, *df* of error variance). This parameter is used to measure the magnitude of the treatment effect. It indicates how far the alternative hypothesis distribution is from the null hypothesis distribution. Since it is possible to describe this scenario as a function of the effect size (*f*^2^) and *df*, everything can be rewritten as an *F* test that involves the λ using the following equation [15]:




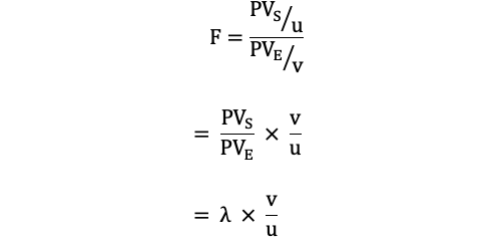




Where PV_S_ represents the proportion of Y (ie, a quantitative dependent variable) variance accounted for by some source (S) in the sample, and PV_E_ represents the proportion of error variance. Finally, the sample size has been calculated as a function of the unknown λ values in terms of the given power (1 – β), u and v values under the significance criteria of .01 and .05, respectively [16].

To account for multiplicity in answering, the sample size was adjusted by incorporating the design effect by considering the intraclass correlation (ICC) and average cohort size (m).

n_adjusted_ = n × {1 + (m – 1) × ICC}

The sample size calculation was based on a noncentral *F* distribution, incorporating the noncentrality parameter (λ) to account for the expected effect sizes. The choice of λ was informed by prior studies on the relationship between smoking behaviors, lifestyle habits, and transitions to ATNPs. Specifically, effect sizes were estimated based on the existing literature examining behavioral and health-related changes associated with smoking transitions. Given the variability in expected effects across different lifestyle factors, a conservative approach was taken in setting λ values to ensure adequate statistical power while minimizing the risk of type 2 errors. Sensitivity analyses were also conducted to assess the robustness of the sample size determination under different assumptions regarding effect sizes and ICC. These considerations strengthen the validity of our study design and ensure that the sample is sufficiently powered to detect meaningful associations.

Therefore, the final sample size is presented in Table 1, with a final choice of 9171 responses, corresponding to a reasonable and common ICC value for such situations.

**Table 1:** Required sample sizes across various scenarios based on ICC, power (1 – β), and other model parameters using the Shiny application [17].

ICC^a^	m^b^	(1 − β)^c^	u^d^	v^e^	*f* ^2f^	λ^g^	Sample size, n
0.1	200	0.8	30	Inf^h^	0.2	29	3031
0.15	200	0.8	30	Inf	0.2	29	4473
0.15	250	0.8	30	120	0.2	29	5561
0.25	200	0.8	24	Inf	0.2	22.5	5709
0.2	250	0.8	28	Inf	0.2	22.5	5715
0.15	300	0.8	30	Inf	0.2	29	6648
0.25	200	0.8	30	Inf	0.2	29	7359
0.2	350	0.8	20	Inf	0.2	21	7434
0.2	250	0.8	20	120	0.15	23.7	8026
0.25	250	0.8	30	Inf	0.2	29	9171^i^
0.2	500	0.9	20	Inf	0.25	26.1	10,524
0.3	350	0.8	20	Inf	0.2	21	11,098
0.3	250	0.8	20	120	0.15	23.7	11,961
0.4	300	0.8	20	120	0.2	21	12,663
0.3	500	0.9	20	Inf	0.25	26.1	15,733
0.5	300	0.8	20	120	0.2	21	15,802

^a^ICC: intraclass correlation coefficient.

^b^m: average cluster size.

^c^(1 – β): statistical power.

^d^u: *df* for the numerator (number of predictors).

^e^v: *df* for the denominator (residual error).

^f^*f*^2^: Cohen effect size index.

^g^λ: noncentrality parameter.

^h^Inf: infinity.

^i^Final sample size used in the study protocol based on the most realistic and conservative assumptions regarding ICC, effect size (*f*^2^), and power. This combination (ICC=0.25, m=250, *f*^2^=0.2, power=0.80) reflects expected values derived from previous smoking behavior studies in similar populations.

### Questionnaire

#### Overview

The questionnaire has been collaboratively developed with a team of experts from the universities of Torino, Padova, and Ferrara, encompassing expertise in areas such as nutrition, biostatistics, data management, and related disciplines. To ensure clarity and comprehensibility, the development process involved interviews and focus groups with smokers, nonsmokers, and former smokers, focusing on both the overall questionnaire and single-term comprehension and interpretation.

The survey is available on the MINERVA project website [18], where ongoing information (eg, the number of participants enrolled and their age and sex) is updated daily.

The questionnaire is organized into various sections. The first section covers sociodemographic details, including information on education, employment status, income, and the number of children and adults in the household.

The questionnaire also addresses lifestyle habits, such as sports and activities and sleep habits. Another section covers information on dietary behaviors, with questions about meal and snack frequency; fruit, vegetable, and fast-food consumption; and alcohol habits. Finally, one section asks about smoking habits, including the frequency of smoking, past smoking habits, and any smoking withdrawals. The survey also examines feelings after smoking. Additionally, a section is dedicated to understanding participants’ attitudes and behaviors toward newer nicotine and tobacco products. This includes knowledge and use of specific products, along with habits associated with these emerging products [19]. The questionnaire items regarding the urge to smoke are adapted from the Brief Questionnaire of Smoking Urges [20]. Furthermore, questions on behavioral intention are derived and modified from McCaffrey et al’s [21] study on the development and validation of behavioral intention measures for an e-vapor product, covering intentions to try, use, dual use, and switch. Lastly, the evaluation of health habits is based on the Sport, Movement, Eating Habits, Relationships, and Technologies (SMART) questionnaire [22].

#### Pilot-Testing and Refinement

The first draft of the questionnaire underwent a pilot test with 12 Italian participants. The sample was characterized by a high degree of heterogeneity regarding the distribution of responders according to gender, age, income level, residence (urban, suburban, rural), education level, tobacco consumption status (smoker, ex-smoker, nonsmoker), coconsumption of different types of tobacco, and coconsumption of other substances (alcohol, cannabis, etc).

Individual interviews explored participants’ comprehension of key definitions within the questionnaire (eg, smoker, ex-smoker, nonsmoker; cigarettes, electronic cigarettes, heated tobacco products, vaping products; up-switching and down-switching between tobacco products; Multimedia Appendix 1).

Individual interviews explored participant comprehension of key definitions (eg, smoker, ex-smoker, nonsmoker; cigarette types; concepts of up-switching and down-switching). Findings from these interviews were then analyzed in a focus group discussion, leading to a series of modifications summarized in Table 2. Following data collection and analysis, a focus group meeting discussed the interview findings and generated suggestions for refining the final questionnaire. Table 2 describes the summary of findings and proposals for modifications highlighted during both interviews and focus groups.

**Table 2:** Summary of findings from interviews and focus group, and questionnaire modification suggestions.

Findings	Proposals for modifications
Nationality could be misinterpreted when the aim is to stratify questionnaire answers	Add country of birth, citizenship, and residence
The open question related to family composition could be difficult to analyze	Modify and add questions related to different relatives (and frequencies), and ask if they smoke. Add a question related to family’s annual income
Nonpublic employee	Change to private employee
Unemployed	Add a question related to identifying who is actively seeking employment and who is not
Attitude toward smoking is missing	Add questions related to social acceptance of smoking, happiness related to smoking or nonsmoking, if the participant would like to quit smoking, if he/she has already tried to quit smoking
Regarding health habits, the survey asks which sport he/she is practicing	This part of the survey should ask at the beginning if the participant is practicing any sport and any agonistic sport Add to the question a specification related to the current days Add multiple answers and revise from open question to closed question
Some questions in the lifestyle habit section of the survey seem related only to young participants or students	Rethink and modify accordingly the questions
There is a section related to sport and the next section is devoted to physical activity	The survey section related to physical activity should add a definition and the differences between sport and physical activity
The response options regarding the frequency of physical activity seem to report frequencies that are too low	Add more answers considering more frequent options
Regarding transportation, something is missing	Add the number of steps recorded in a day and the use of stairs compared to elevators
Regarding the question related to beverages, something is missing	Consider adding wine, beer, aperitifs, alcohols, and only water
Related to sleep attitudes, the options available are not suitable for those working in shift	Modify the options
About the influence of sleep on physical conditions, a 4-point Likert may not be able to capture the differences between participants	Consider using a visual analog scale

Following these refinements, a second pilot test was conducted to validate the adjustments and ensure logical coherence, branching accuracy, and cultural appropriateness. Table 3 summarizes additional modifications implemented based on the second round of feedback.

**Table 3:** Summary of findings provided by questionnaire pilot test and suggested modifications.

Findings	Proposals for modifications
Regarding the required description of the family of the participant, it may not be clear if the participant should be included	In the question, it should be specified that the participant is included in the description
Some ways of consuming tobacco/nicotine are missing, both conventional and unconventional	Revise the list of tobacco/nicotine products and add sachets
Some branching logic is not working properly	Recheck branching logics
The order of questions proposed in the survey can be quite confusing because the leading theme is changing too often	Some questions should be moved through the survey to place them together according to the different themes/sections
Unconventional employment is missing	Some kinds of unconventional employment should be added
Discussion on adding or removing ethnicity/race	Remove ethnicity and race
Semantic differences between smoking at home and smoking inside your home	The survey focuses on smoking at home
No data is collected on feelings after smoking	A questionnaire on smoking urges has been added
The survey should also address the perception of participants about the consequences	The Short Form Vaping Consequences Questionnaire has been added
The survey should try to measure the level of dependency of participants	Penn State Electronic Cigarette Dependence Index
The survey is not able to check if the participant is reading and accepting GDPR^a^ information and if the participant was the minimum age for participating	Add check about reading and accepting GDPR information and the minimum age for participating
The description of different ways of consuming tobacco/nicotine can be improved by providing pictures	Add pictures
Waterpipe is missing	Add waterpipe

^a^GDPR: General Data Protection Regulation.

The development of the final questionnaire involved a process comprising the following steps:

1. Compilation: gathering pertinent questionnaires for each section
2. Translation: translating all questions into Italian
3. Curation: selecting only the pertinent questions for each section
4. Focus group: conducting a focus group session
5. Refinement: fine-tuning the questionnaire based on insights derived from the focus group
6. Testing: assessing the prefinal version
7. Further refinement: tweaking the final version based on testing results
8. Backward translation: conducting a backward translation to English of the entire questionnaire

**Figure 1:**
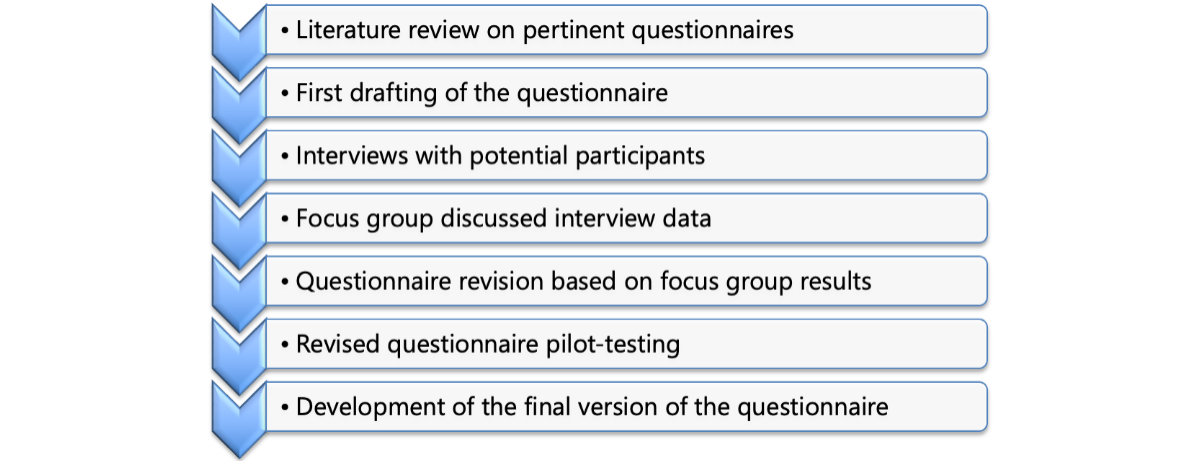
The phases of questionnaire development.

This systematic approach ensures the comprehensiveness, linguistic appropriateness, and effectiveness of the questionnaire, with iterative refinement occurring (Figure 1).

#### Cultural Adaptation and Cross-Country Comparability

Given the diverse cultural and regulatory landscapes of the participating Mediterranean countries (Italy, Greece, and Turkey), our study acknowledges the potential influence of cultural differences on smoking behaviors and attitudes toward ATNPs. To ensure cultural sensitivity and comparability across populations, linguistic and contextual adaptations were made through multistage expert reviews, focus groups, and interviews involving participants from different cultural backgrounds. Additionally, stratified analyses will be conducted to examine potential variations in smoking behaviors and attitudes across cultural and geographic subgroups. These measures aim to enhance the accuracy and inclusiveness of our findings, providing a comprehensive understanding of the interplay between cultural factors and smoking transitions.

### Statistical Analysis

Baseline and demographic characteristics of the study participants will be summarized using medians (IQRs) for continuous variables and absolute and relative frequencies for categorical ones. To examine the associations between smoking habits, dietary patterns, and lifestyle behaviors, univariable and multivariable analyses will be conducted using generalized linear models, with the appropriate distribution selected based on the outcome variables. Confounding variables such as age, gender, and socioeconomic status will be adjusted for using multivariable regression models, informed by prior literature. Interaction effects will be evaluated through interaction terms within models and stratified analyses across demographic subgroups, with likelihood ratio tests used to determine their significance. All analyses will be carried out using R software (R Foundation for Statistical Computing).

### Data Collection and Management

Data will be collected using a structured questionnaire administered via LimeSurvey [23]. Once the data is collected, it will be exported in a format suitable for statistical analysis. Data will be securely stored on a private password-protected server with access limited to authorized personnel only. To ensure data security, the server will use encryption protocols and role-based access controls, preventing unauthorized modifications or downloads. Data will be stored in servers located in Western Europe; the data will not be transferred outside of Europe.

Regular backups of the dataset will be made to prevent any loss of data. All data management procedures will comply with GDPR and other applicable data protection and privacy regulations.

Given the longitudinal nature of the study, participant retention and potential changes in behavior over time are key considerations. To address the issue of dropouts, participants who consent to follow-up will be recontacted biannually, with reminders sent by email and social media to encourage continued participation.

## Results

The MINERVA study was initiated in June 2023, and data collection is currently ongoing. As of January 2025, the study has enrolled 7535 participants in Italy, with 4862 consenting to follow-up. Preliminary analyses are underway, focusing on sociodemographic characteristics, smoking behaviors, and economic factors. Follow-up assessments will be conducted every 6 months, with results expected to be published at the end of 2025.

## Discussion

This protocol outlines the methodology for investigating the association between smoking behaviors, lifestyle factors, and dietary habits, with a particular focus on transitions between conventional tobacco products and ATNPs. By using a longitudinal multicountry approach, the study aims to provide valuable insights into behavioral patterns and factors influencing smoking transitions.

The existing literature highlights the complex interplay between smoking cessation and sociodemographic characteristics. However, gaps remain in understanding how lifestyle factors, particularly dietary habits, influence the use of ATNPs and long-term smoking behaviors across diverse populations [24]. Compared to previous cohort studies, this approach improves the ability to capture trends over time and identify predictors of change in smoking behavior.

A key strength of this study is the use of social media–based recruitment and snowball sampling, which facilitates access to diverse populations while ensuring cost-effective and scalable data collection. However, selection bias is a potential limitation, as individuals with higher engagement in digital platforms may be overrepresented. To mitigate this, recruitment strategies will aim to reach a broad demographic, and follow-up analyses will assess the generalizability of findings. Furthermore, self-reported data can introduce recall or social desirability biases; however, the longitudinal design helps to track changes within individuals over time, reducing potential inaccuracies.

Understanding the lifestyle dangers associated with both traditional smoking and ATNPs is essential for public health efforts. Ongoing research is needed to further assess the health risks of ATNPs and to inform evidence-based policies and interventions. Although less harmful than traditional smoking, ATNPs are not without potential risks, and it is important to continue research efforts to fully understand the long-term health implications of ATNPs and to inform evidence-based policies and interventions [11].

## Appendix

Multimedia Appendix 1 Interview guide for questionnaire development.

## Abbreviations

ATNP: alternative tobacco and nicotine product
ICC: intraclass correlation
MINERVA: My Changing Lifestyles Our Research and Everyone Health
SMART: Sport, Movement, Eating Habits, Relationships, and Technologies

## Data Availability

The datasets analyzed during this study will be available from the corresponding author upon reasonable request.
