# Supplementary material for: Attitudes Toward Alternative Tobacco and Nicotine Products and Their Association With Lifestyle Habits: Protocol for the MINERVA International Observational Cohort Study
Source: JMIR Res Protoc. 2025 Oct 14;14:e67163. doi: 10.2196/67163 (PMC12569482; doi:10.2196/67163)
Supplement: Multimedia Appendix 1 [file resprot_v14i1e67163_app1.pdf]

Gregori et al. 2025 Assessing attitudes towards alternative tobacco and nicotine products and their association with lifestyle habits: protocol for the MINERVA (My changINg lifEstyles our Research and eVeryone heAlth) international observational cohort study
Appendix 1

Interview Guide for Questionnaire Development

Introduction:
Thank you for participating in this interview. The purpose of this interview is to gather your perspectives on smoking-related habits, terminology, and potential questions for inclusion in a public health questionnaire. This questionnaire will be used to collect data on lifestyle habits, including diet, physical activity, and tobacco consumption. The final questionnaire will be distributed electronically to a target population to support public health research.

This interview consists of two main parts:

1. Definitions: We will ask you to define certain terms related to smoking behavior, similar to dictionary definitions. The goal is to ensure that the terms used in the questionnaire have a clear and shared meaning.
2. Question Comprehension: We will present you with draft questions and response options intended for the questionnaire. We will ask for your feedback on their clarity, relevance, and whether any questions might be perceived as intrusive or difficult to answer.

Part 1: Definitions of Key Terms

1. How would you define a "smoker"?

- Would this definition include occasional smokers?
- Should the definition be restricted to cigarette use, or should it include cigars, pipes, and electronic cigarettes?

2. How would you define an "ex-smoker"?

- Does this term apply to anyone who has quit smoking, regardless of how long ago they stopped?
- Is there a specific time frame (e.g., six months, one year) that would be appropriate for considering someone an ex-smoker?

3. How would you define an "occasional smoker"?

- Would this term apply to individuals who smoke only in social settings or irregularly?
- Would a person who does not buy cigarettes but smokes when offered by others be considered an occasional smoker?

4. How would you describe a "cigarette"?

- What elements are necessary for a product to be considered a cigarette (e.g., filter, rolling paper, tobacco)?

- Would you consider hand-rolled cigarettes to be the same as pre-packaged ones?

5. How would you describe an "electronic cigarette"?

- What are the distinguishing features of an e-cigarette compared to a traditional cigarette?
- Would you differentiate between e-cigarettes that use liquid vaporization and those that heat tobacco?
- Do you think e-cigarettes contain tobacco or other harmful substances?

6. How would you describe the transition from one form of smoking to another (e.g., from traditional cigarettes to e-cigarettes or heated tobacco products)?

- Would you consider this transition as an effort to quit smoking or an alternative way to continue smoking?
- Do you think people use e-cigarettes and heated tobacco products in addition to traditional cigarettes rather than replacing them?

Part 2: Evaluation of Draft Questionnaire Questions

For each of the following questions, we ask you to consider:

- Clarity: Is the question easy to understand?
- Appropriateness: Does the question seem relevant and suitable for the target population?
- Privacy Concerns: Would respondents feel comfortable answering this question?

Provide the latest version of the questionnaire.
